# Pregnancy-Induced Isolated Severe Vaginal Varicosities: A Case Report and Literature Review

**DOI:** 10.1055/a-2765-7948

**Published:** 2026-01-14

**Authors:** Hanna Brancaccio, Itishree Panda, Isabelle Crary, Asha Rijhsinghani

**Affiliations:** 1 Department of Medicine, Rowan-Virtua School of Osteopathic Medicine, Stratford, New Jersey, United States; 2 Department of Obstetrics and Gynecology, Srimanta Sankardeva University of Health Sciences, Guwahati, Assam, India; 3 Department of Obstetrics and Gynecology, University of Washington, Seattle, Seattle, Washington, United States; 4 Morristown Medical Center, Morristown, New Jersey, United State

**Keywords:** prolapsing vaginal varicosities, pregnancy, obstetrics

## Abstract

**Objective:**

During pregnancy, total blood volume increases by about 40%. As gestation advances, blood volume to the pelvic region increases significantly. Due to the enlarging uterus, the venous return from the lower body decreases with advancing gestational age. The pooling and stasis of blood in the lower body can lead to the formation of varices, affecting the lower extremity, vulva, and vaginal regions. Varicosities in the lower extremities and vulva are not uncommon and often asymptomatic. Vulvar varicosities are more common in women with varicosities in the lower extremities. On the other hand, vaginal varicosities are extremely rare and mentioned only in case reports. Although asymptomatic, vaginal varicosities could become a concern for hemorrhage risk during vaginal delivery. There is little information in the current medical literature about the diagnosis and management of large vaginal varicosities during pregnancy.

**Study:**

Design We present a case of a primigravida with very large prolapsing vaginal varicosities that presented as a large external mass at 36 weeks of gestation.

**Results:**

The patient was managed expectantly during the antenatal period. She was delivered via an elective scheduled cesarean section. An almost complete resolution of the vaginal varices was noted at 6 hours postoperatively, with complete resolution reported on postoperative day 22.

**Conclusion:**

Due to the rarity of the condition, we hope to add our experience to the literature.

## Introduction

During pregnancy, many physiologic hemodynamic changes are known to occur. The total blood volume increases by about 40 to 50%, and blood flow to the lower half of the body, as well as the pelvis, increases in parallel. 1 This, in turn, leads to venous congestion, which predisposes the patient to varicosities. Varicosities in the lower extremities as well as the vulva are not uncommon, and when these occur, they are usually seen after 12 to 26 weeks of gestation. In the majority of patients, these largely resolve spontaneously by 6 weeks postpartum. 2 3 Vaginal varicosities are extremely rare, and the associated risks have not been clearly defined. Concerns of vaginal variceal rupture and hemorrhage during vaginal childbirth may lead practitioners to consider delivery via cesarean section. There is limited literature regarding the outcomes of such an approach. 2 4 5 We present the management and outcome of a case of extremely large vaginal varicosities, presenting as a large prolapsing mass during the antenatal period. The patient's written consent was obtained from one of the authors.

## Case

A 35-year-old, healthy primigravid female, with no history of chronic illness, no prior surgical history, and a history only of tobacco chewing, presented to the health center at 36 weeks of gestation with complaints of discomfort in the lower pelvic region. She was experiencing a sensation of something protruding from her introitus for the past month.

Prior to pregnancy, the patient had no history of gynecological problems. Her menarche was at age 15 years, and she had a history of mild dysmenorrhea during her menstrual cycles. She denied a history of any gynecologic diseases, and her last STI screening in the current pregnancy was negative. Significantly, her job required her to be on her feet for the majority of the day.

One month prior to arrival at the health center, the patient reported feeling discomfort in her lower abdominopelvic region, followed by a sensation of protrusion of a mass from her vagina for 2 to 3 weeks. She denied vaginal discharge, dyspareunia, and dysuria. Though present throughout the day, the symptoms were more pronounced during walking and prolonged standing. Due to the persistence of the symptoms, she sought medical attention.

**Fig. 1:**
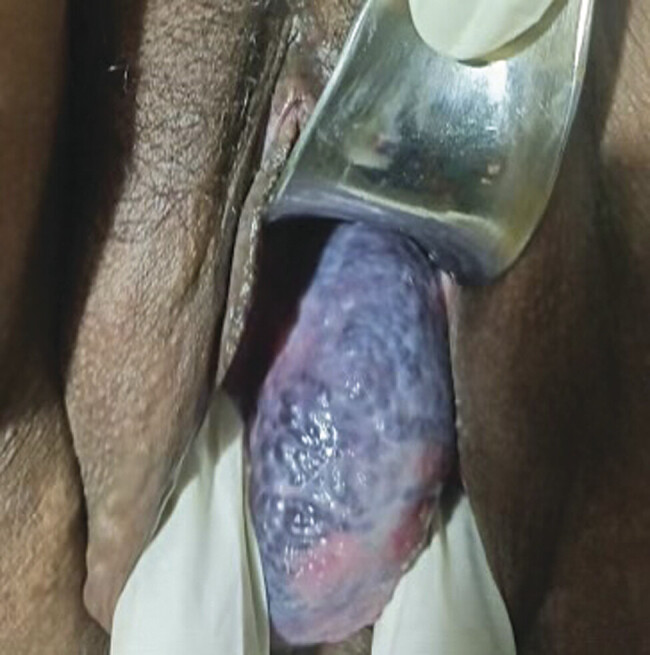
Presenting symptoms of protrusion of a mass from the vagina, in the third trimester.

On pelvic exam, a 6 cm × 4 cm mass of dilated tortuous blood vessels, contained in a thin-walled sac, was visualized protruding from the introitus. On speculum exam, it was seen to arise from the left lateral vaginal sidewall ( Fig. 1 ). The mass was dark blue with contents that were prominent blood vessels. On palpation, it was soft in consistency and did not bleed upon manipulation. There were no other dilated vessels noted on the vulva; however, significant superficial varicosities were visualized on the left medial thigh just superior to the knee.

The patient was diagnosed with large prolapsing vaginal varicosities, and due to suspected stasis of the blood in these vessels, she was started on a prophylactic anticoagulation dose of 40 mg/day of low molecular weight heparin (LMWH). In addition, she was also recommended to use compression stockings in both lower limbs, keep the lower limbs elevated while sleeping, to avoid straining and standing for prolonged periods of time. The patient was also diagnosed with iron deficiency anemia, with a hemoglobin of 8.2 g/dL, for which she was treated with parenteral iron sucrose. To prevent hemorrhage from rupture of the varicosities, an elective cesarean section was recommended at 39 weeks of gestation.

**Fig. 2:**
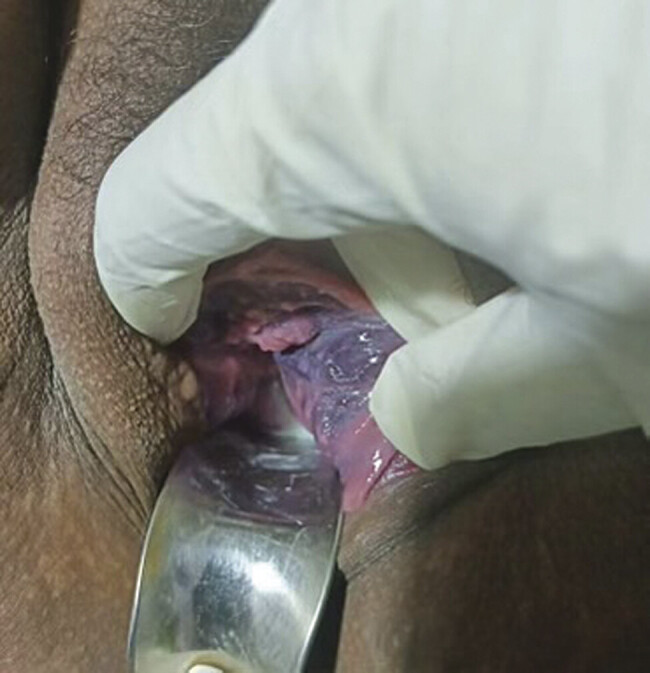
Six hours postoperative, near complete resolution of the vaginal varicosities, with only slight bluish discoloration of the left lateral vaginal wall.

During the elective cesarean section, intraoperatively, the uterine vessels were noted to be significantly engorged and tortuous. A healthy female infant weighing 2.9 kg was delivered without complications. Mild postpartum hemorrhage due to uterine atony was managed conservatively. Total blood loss was estimated at 1,100 mL. Six hours postoperatively, a pelvic exam was performed. The vaginal varices were no longer noted outside the introitus. A repeat speculum examination was performed, which demonstrated near complete resolution of the vaginal varicosities, with only slight bluish discoloration of the left lateral vaginal side wall ( Fig. 2 ). Three weeks later, the patient returned for postoperative check and complete resolution of the varicosities was noted, including resolution of the bluish discoloration of the left vaginal side wall. Due to the complete resolution of the vaginal varicosities, no further treatment was planned.

## Discussion

Pregnancy causes a myriad of physiologic changes, including an increase in blood volume and vena caval compression by the enlarging uterus, which progressively increase as the gestation advances. Acquired vascular refractoriness to angiotensin II also causes blood vessel dilation and decreased venous pressure. These changes increase venous congestion, which predisposes pregnant individuals to varicosities in the lower half of the body. 6 7 While vulvar varicosities are reported to arise in up to 4% of pregnancies, the incidence and severity of vaginal varices have not been studied due to sparse published literature. 6 8 9 Vaginal varicosities are believed to be rare due to the numerous outlets for venous flow via the venous plexus. 10 In the nonpregnant patient, etiologies have been reported and linked to portal hypertension and Klippel–Trenaunay syndrome. 10 11 12 13 During pregnancy, heavy bleeding has been reported, mimicking placenta previa. 14 Surgical management of bleeding vaginal varicosities has been reported, though the optimal management has yet to be defined. 8 14

In this case, the patient presented at 36 weeks of gestation with large vaginal varicosities protruding from the introitus. Our main concerns were the possibility of thrombosis during the pregnancy and injury during delivery, with potential for heavy bleeding and related complications. Due to these concerns and limited guidance from previously published reports, we chose to manage the patient with prophylactic doses of LMWH during the pregnancy to prevent antenatal thrombosis. Additionally, we planned a scheduled cesarean delivery at 39 weeks to avoid hemorrhage from inadvertent rupture of the varices during a vaginal delivery, keeping in mind that the patient had severe anemia. It was pleasantly surprising for us to note the almost complete resolution of the vaginal varices, 6 hours postoperatively.

In a prior case report, where heavy bleeding was encountered, authors have suggested consideration of various possible treatment options that include ligation, cauterization of the vessels, laser photocoagulation, and topical treatment with astringent creams such as phenylephrine and hydrochloride cream, in cases that do not spontaneously resolve. 14 These may be attempted either during pregnancy if bleeding is encountered or postdelivery. In our patient, due to the complete resolution at 3 weeks postpartum, we did not perform any therapeutic procedure for treatment of the condition. Since we did not attempt any treatment during the inter-pregnancy interval, we cannot address the possibility of recurrence of the condition in future pregnancies.

## Conclusion

The diagnosis of vaginal varicosities during pregnancy may not become evident until late in the third trimester. During pregnancy, there are no current guidelines for the management of the condition due to a lack of published literature on this very rare condition. Our patient presented at 36 weeks with new symptoms due to the extremely large vaginal varicosities that protruded out of the vagina. Due to the rarity of the condition and lack of published data, we proceeded with the thromboprophylaxis to prevent thrombosis, a leading cause of maternal morbidity and mortality due to the hypercoagulable state induced by pregnancy and increased venous stasis, as well as elective cesarean delivery, and the outcome was good.
